# A Strategy to Simultaneously Cure Type 1 Diabetes and Diabetic Nephropathy by Transplant of Composite Islet-Kidney Grafts

**DOI:** 10.3389/fendo.2021.632605

**Published:** 2021-05-12

**Authors:** Thomas Pomposelli, Christian Schuetz, Ping Wang, Kazuhiko Yamada

**Affiliations:** ^1^ Columbia Center for Translational Immunology, Department of Medicine, Columbia University Irving Medical Center, New York, NY, United States; ^2^ Department of Surgery, Columbia University Irving Medical Center, New York, NY, United States; ^3^ Precision Health Program, Michigan State University, East Lansing, MI, United States; ^4^ Department of Radiology, College of Human Medicine, Michigan State University, East Lansing, MI, United States

**Keywords:** composite graft, kidney, islets, transplantation, diabetes

## Abstract

In recent years islet cell transplant has proven itself to be a viable clinical option for a select group of diabetic patients. Graft loss after transplant however continues to hinder the long-term success of the procedure. Transplanting the islets as a pre-vascularized composite islet-kidney graft has emerged as a relevant solution. Much groundbreaking research has been done utilizing this model in conjunction with strategies aimed towards islet cell survival and prolongation of function in the host. Transplanting the islet cells as a prevascularized graft under the capsule of the donor kidney as a composite islet-kidney graft has been shown to provide long term durable blood glucose control in large animal studies by limiting graft apoptosis as well as providing a physical barrier against the host immune response. While promising, this technique is limited by long term immunosuppression requirements of the host with its well-known adverse sequelae. Research into tolerance inducing strategies of the host to the allogeneic and xenogeneic islet-kidney graft has shown much promise in the avoidance of long-term immunosuppression. In addition, utilizing xenogeneic tissue grafts could provide a near-limitless supply of organs. The islet-kidney model could provide a durable and long-term cure for diabetes. Here we summarize the most recent data, as well as groundbreaking strategies to avoid long term immunosuppression and promote graft acceptance.

## Population of Patients With Type 1 Diabetes (T1DM) and End-Stage Renal Disease (ESRD)

Type 1 diabetes mellitus (T1DM) is a chronic autoimmune disease characterized by T-cell mediated destruction of pancreatic β-cells, leading to insulin deficiency and hyperglycemia. The classic symptoms of polyuria, polydipsia, weight loss and fatigue often manifest themselves at a young age, however T1D can clinically manifest at any age (1–3). Diabetes is also the leading cause of chronic kidney disease (CKD) and end stage renal disease (ESRD) in the United States as well as worldwide (4, 5).

Diabetic nephropathy is a major cause of morbidity and mortality among patients with diabetes, and its prevalence among children has been steadily increasing (6). While the adjusted 5-year survival in people with diabetes starting on hemodialysis is 29%, diabetic kidney recipients of either a deceased or living donor was 75% and 83%, respectively (7). The pathogenesis and progression to diabetic nephropathy involves a complex interplay between metabolic and hemodynamic factors, namely hyperglycemia and hypertension (8). This ultimately leads to both functional and structural changes in the kidney that ultimately leads to nephropathy, which is characterized by increasing albuminuria and declining renal function. Of note, CKD in people with DM is not limited to the pathophysiology known as diabetic nephropathy but can also be due to non-diabetic kidney disease, due to risk factors independent of hyperglycemia, or an overlap of both pathologies (9).

In the early 90’s The Diabetes Control and Complications Trial (DCCT) and the UK prospective diabetes study (UKPDS) demonstrated that maintaining blood glucose as close to physiologically normal would halt this progression to nephropathy, and in so doing determine whether the complications of T1DM could be delayed or outright prevented (10, 11). After a 30 year follow up the study convincingly demonstrated that maintaining glycemia as close to the nondiabetic range reduced all of the microvascular and cardiovascular complications of diabetes, including a reduction in the risk of developing ESRD by 50% (12, 13). DCCT also showed that retention of residual endogenous insulin secretion (defined by residual C-peptide secretion) was associated with a lower frequency of hypoglycemia and microvascular complications, including nephropathy (14, 15).

It is however challenging to achieve physiological glucose control for various limitations of intensive insulin therapy regimens, with the risk of hypoglycemia recognized as a major hurdle (16). The incidence of ESRD in patients with T1DM is not well defined and ranges widely in the literature as well as a reduction in recent decades due to improvements in insulin therapy and renal care (8, 17, 18). To date the only true cure for diabetes and ESRD remains whole pancreas and kidney transplant (3, 19, 20). Upon review of the data from the International Pancreas Registry, while outcomes of pancreas and kidney transplant have improved, achieving long-term survival and graft function remains far from ideal. Between 2004 and 2008 the most common pancreas transplant was a combined pancreas/kidney transplant (SPK), as it provides a physiological means of achieving normoglycemia while rendering ESRD patients free of dialysis (21).

The five year graft survival rates are currently 73% for simultaneous pancreas and kidney, 65% for pancreas after kidney, and 53% for pancreas alone transplant. Furthermore, pancreas transplant itself is accompanied with an estimated 10 – 20% complication rate including graft thrombosis, enteric leak, rejection or chronic pancreatitis (22).

## Islet Transplantation and the Importance of Pre-Vascularization

In recent years, the clinical applicability of pancreatic islet cell transplant has gained momentum as a possible alternative to pancreas transplant due to the relative safety of the procedure and approaching results similar to SPK (21, 23, 24). Allogeneic islet transplantation is indicated for patients with type 1 diabetes complicated by severe hypoglycemia and hypoglycemia unawareness or marked glycemic lability, or for patients already on chronic immunosuppression in support of a kidney transplant. In such patients, islet transplantation has been proven effective in restoring hypoglycemia awareness and abolishing severe hypoglycemic episodes (3, 25). Furthermore, islet transplantation appears to be a valid option for patients with severe, unstable T1D who are not responding to intensive insulin therapy (26). In addition, recent observational evidence suggested that islet transplantation is not associated with increased mortality regardless of the use of long-term immunosuppressive therapy (27).

The transplant itself involves harvesting a donor pancreas, and isolating the primary islet cell clusters, and the purified islets are then injected intra-portally into a diabetic recipient wherein the cells engraft within the liver parenchyma (28). One of the most successful demonstrations of islet cell transplant was published in 2000 by the Edmonton group, in which they were able to achieve insulin independence in 7 out of 7 T1DM patients treated with islet cell transplant (29). A long-term follow up of the patients in the study however revealed that approximately 75% of the recipients required exogenous insulin at 2 years after their transplant (30). Since the publication of this landmark study, specialized centers now expect a 1‐year islet allograft survival (defined by fasting basal C‐peptide level ≥0.5 ng/mL) rate of 41%, and 11% of the recipients can expect to be insulin independent at 1 year post‐transplant. While this is certainly a vast improvement, more studies need to be done to make islet transplant a durable clinical option (31).

The reasons for the limited long-term success of islet cell transplant is multifactorial, and include multiple immunological and non-immunological factors including allograft rejection, recurrence of autoimmunity (32–34), and immunosuppressant toxicity (35, 36). In an attempt to ascertain if the immunogenic effects on islet cells after transplant could be avoided, our group has studied if simultaneous transplantation of kidney grafts with islet cell infusion facilitated allogeneic islets to engraft in pig allogeneic and cynomolgus monkey allogeneic transplant models. In these studies, all recipient animals underwent native nephrectomies and induced diabetes by total pancreatectomy (pigs) or streptozotocin injection (cynomolgus moneys). Unlike kidney models, induction of tolerance of islets were unsuccessful using regimens that successfully induce tolerance of allogeneic kidneys (37, 38). Another well documented barrier to the survival of islet cells is the instant blood-mediated inflammatory reaction (IBMIR) (39). This is the inflammatory reaction seen after islets come into contact with the recipient blood, and it is characterized by a general inflammatory response leading to islet apoptosis and graft loss. It has also been shown that exposure of the islets directly to high concentrations of glucose also negatively affects their function (40), although we could not confirm this in our models (41). Following infusion, the islet cells are unique from other grafts in that they are free floating in the circulation, and as such deprived of a vascular supply of essential oxygen and nutrients, which further leads to an inflammatory response in the cells as well as apoptosis. Despite these obstacles, islet cell transplant remains a very attractive surgical cure for diabetes due to the relative safety of the procedure itself.

## Composite Islet-Kidney Graft: New Concept to Transplant Donor Islets as a Vascularized Tissue as a Part of the Donor Kidney

A promising and potential answer to overcome the problems of direct islet cell transplant involves the generation of a composite islet-kidney graft. Experimental and clinical observations support the generalization that vascularized organs have prolonged graft function over non-vascularized grafts, In addition, while vascularized grafts are relatively tolerogenic (42–45), skin and tissue grafts are relatively immunogenic, probably because they sensitize the host by releasing cells or antigens into the lymphatic system (46). Previous studies of allogeneic islet transplantation, usually by injection into the portal vein, have had limited success, not only because of the shortage of available islets but also because of failure of these islets to engraft or to remain functional (23). Although the “Edmonton Protocol” originally showed good survival of islet transplants at one year (47), by 5 years, in a much larger series of patients, only 10% of patients remained insulin independent (29, 30). In contrast, whole organ pancreas transplantation, although prone to serious surgical complications, has led to improved results when technically successful (48–50).

**Figure 1:**
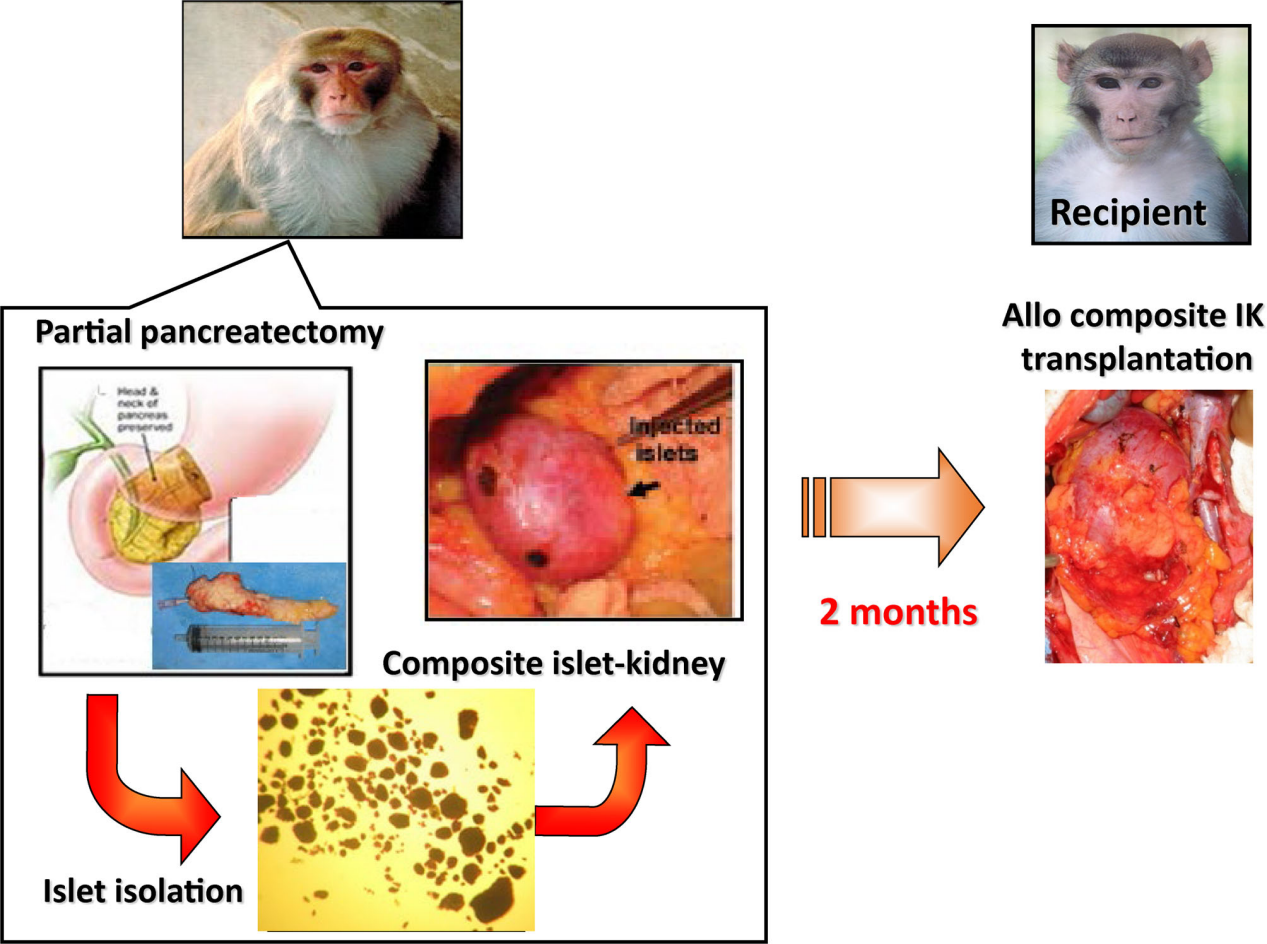
Schematic diagram of preparation of a composite islet-kidney (I-K) graft in a donor and allogeneic composite I-K transplantation.

A major reason for this difference is the fact that tissue grafts, which require the establishment of a new vascular supply in order to survive, are much more susceptible than whole organ grafts to failure from nonspecific causes, such as inadequate oxygen or nutrient supply. We have reasoned that this survival advantage might be extended to islets by transplanting them as part of a composite vascularized graft. The benefit of this “Trojan-horse” approach for diabetic patients is really two fold, in that it would provide a durable long term cure for diabetes through a relatively low risk surgical procedure, as well as curing or preventing the subsequent ESRD that often characterizes diabetes. Composite I-K transplantation could be particularly relevant for the population of juvenile diabetic patients that receive living-related kidney-transplants for ESRD. On this basis, much work has been done on large animals to develop the techniques required for constructing composite “islet-kidneys” (I-K) (38, 51, 52) (**Figure 1**).

A proof of concept study was first published in 2002 in which composite I-K’s were prepared in a miniature swine model (38, 53), and this strategy was extended to nonhuman primate models (51, 52) (**Figure 1**). Islet isolation was performed *via* partial pancreatectomy (70% distal pancreatectomy) and the islets were purified from excised pancreatic tissue by enzymatic digestion and discontinuous density gradient purification. Isolated islets were cultured for 2 days before transplant, thereafter they were then injected under the renal capsule of the same donors. Two to 3 months later, recipients of the composite I-K were induced diabetes (IDDM) surgically *via* a total pancreatectomy. All recipients were hyperglycemic before their transplant, with blood glucose > 300. 7 days following total pancreatectomy, the recipients underwent total bilateral nephrectomy followed by orthotopic renal transplant of the I-K graft. As controls, IDDM animals in the non-vascularized islet group received allogeneic islets directly implanted under recipient’s renal capsule (51). After I-K transplantation, blood glucose levels fell immediately to normal levels (<120 mg/dl) by post-operative day (POD) 1. I-K grafts were permanently accepted, and recipients maintained normoglycemia with normal creatinine and renal function. Of note, recipients in this study received roughly 10,000 IEQ/kg in both the I-K group and the intra-portal infusion group. This is noteworthy because this islet yield was obtained from a single donor in a non-lethal partial pancreatectomy. In contrast, recipients of non-vascularized islets had increasing blood glucose from POD 1, and the islet grafts were rejected based on histological criteria by POD 23 even in MHC-matched donor-recipient pairs. The study was extended to transplants across full MHC mismatches (53). All IDDM and both native kidney nephrectomized recipient swine accepted life-supporting IK-grafts and maintained normal blood glucose and serum creatine levels with a short course of calcineurin inhibitor. Moreover, these recipients showed donor-specific unresponsiveness, indicating the occurrence of immune tolerance of both islets and kidneys following I-K transplantation. This study demonstrated that non-vascularized islet transplants isolated from a single donor are not capable of curing surgically induced diabetes, while in contrast a similar number of prevascularized islets within composite I-K grafts can cure surgically induced diabetes.

In order to further test the clinical applicability of this strategy, a study from 2011 was performed in which the function of I-K grafts was directly compared to direct intra-portal islet transplant in a nonhuman primate (NHP) baboon model (51). For this study, the I-K grafts were prepared in donor baboons in a similar fashion after 70 – 80% pancreatectomy, and the islets were injected under the renal capsule. Three of the recipient IDDM and both nephrectomized baboons underwent life-supporting orthotopic I-K transplant, and the other three animals were not host nephrectomized received allogeneic islets directly into intra portal veins or under recipient’s renal capsule. The recipient animals were maintained on an immunosuppressive regimen of tacrolimus and mycophenolate mofetil (MMF), which is a typical regimen used in clinical kidney transplantation and is not islet specific. We demonstrated that recipients of the I-K’s had stable renal function, as well as noted reversal of their hyperglycemia for the duration of the experiment (> 250 days) without need for exogeneous insulin. We also confirmed that islets of composite I-K grafts regulated blood glucose *via* assessment by intravenous glucose tolerance test 3-8 months following transplantation. The different effects of insulin and islet function between infusion through the portal system (intrahepatic islets) vs inferior vena cava (islets in composite I-Ks) may be an interesting issue to clarify, although insulin therapy in clinic is administered subcutaneously or intravenously *via* a peripheral vein.

Notably, these animals received a mean of 14,127 IEQ/kg islets. In contrast, all animals that received direct infusions of islets in greater numbers (19,000; 17,000; 32,000 IEQ/kg, respectively) either into recipients’ livers or under the renal capsule at the day of allogeneic transplantation all went on to require exogeneous insulin and ultimately were sacrificed secondary to complications from hyperglycemia (51). This is an important point for clinical translation of the study, as minimizing harm to the donors is paramount. Therefore, the 70% distal pancreatectomy for composite I-K preparation had proven to not only be safe for the donors but also providing enough islet cell mass to reverse hyperglycemia and a life supporting kidney graft in anephric, diabetic porcine and primate recipients. Additional strategy to minimizing of the extend of donor pancreatectomy for I-K preparation in donors for future clinical trials are discussed in later sections.

## Tolerance Strategy in Islet Transplantation Is Required to Avoid Immunosuppression and Recurrent Autoimmunity

The overall goal of islet transplantation is to provide freedom for patients from both insulin as well as immunosuppression. Although we have reported tolerance of islets and kidney in swine I-K transplant model (53), baboon models that described above used chronic immunosuppression which is a typical regimen used in clinical kidney transplantation (51).

Steps towards clinical application of our composite I-K transplantation to cure diabetic nephropathy, we further extended our studies for the induction of tolerance of composite I-K grafts in NHPs. We have recently reported successful induction of allograft tolerance to both islets and kidneys across a “parent to offspring” haploidentical barrier using the composite I-K grafts in rhesus macaques (52). Composite I-K grafts were accepted in allogeneic recipients that received peripheral blood hematopoietic stem cell transplant (PBHSCTx) with an “Irradiation, T cell depletion, and Cyclosporine A (ITC)” regimen that consisted of a low-dose total body irradiation (100cGy TBI) in combination with transient recipient T-cell depletion and a 45-day course of cyclosporin A (CyA) (52). IDDM and both native kidney nephrectomized recipients accepted both kidney grafts and islets even after the cessation of immunosuppression >200 days. This also provided a proof of concept demonstrating that composite I-K facilitates induction of tolerance of both islets and kidney. Tolerance regimens using mobilized donor cells obtained by leukapheresis facilitated allogeneic composite I-K acceptance, providing “proof of principle” for the IK approach. However, because of fluctuated blood sugar levels in the induction period due to CyA, we are currently optimizing the induction regimen to be clinically applicable for I-K transplantation. Other promising avenues of study to induce tolerance include pharmacologic strategies specifically targeting the toll-like receptor 4 (TLR4), which is a transmembrane receptor that when stimulated induces a pro-inflammatory state and contributes to the maintenance of autoimmune diabetes through the activation of CD4+ T lymphocytes. A recent study demonstrated in a murine model that when TLR4 signaling was inhibited CD4+ T lymphocyte proliferation was inhibited, and the onset of spontaneous diabetes was avoided (54).

## Limitation of Composite I-K Strategy Across Allogeneic Clinical Transplantation

While much success and promising work has been done (38, 51–53), there remains two limitations to I-K transplant as a viable clinical option. The major problem with the composite I-K procedure as a cure for diabetes is the need to establish a vascularized I-K *in vivo* in the donor 1-3 months prior to transplantation, which makes this approach applicable only to living-donor transplants. While composite I-K transplantation eliminates the complications associated with solid organ pancreas transplantation (55, 56) and 70% of partial pancreatectomy did not change blood glucose levels in recipient >360 days in our experimental models (38, 51, 52), risks associated with partial pancreatectomy from living donors is not completely eliminated. Therefore, the safety of the preparation and transplantation of allogeneic I-K may be clinically acceptable if the donors are highly motivated and screened carefully to assure that they are not pre-diabetic.

Another issue is also obtaining a large enough mass of islets to overcome hyperglycemia and maintain normoglycemia in recipients. I-K protocols usually require up to 10,000 IEQ/kg to achieve insulin independence, and direct islet infusion protocols require much more, often necessitating the use of more than a single donor for each recipient (29, 30). This in turn may also increase the risk of allosensitization (57, 58).

### New Strategy to Minimize Pancreas Mass by Preventing Apoptosis of Islets

Several studies have been done that have utilized a variety of strategies aimed at preventing apoptosis in the islet cell graft after transplant (59). Our colleagues have recently synthesized a novel small interfering RNA (siRNA)-nanoparticle probe designed to specifically target the apoptotic-related gene caspase-3. By utilizing small interfering RNA (siRNA) gene therapy, they have demonstrated the protective effects of silencing the pro-apoptotic gene caspase-3 in donor islet cells which resulted in significantly better survival of transplanted islet cells in a murine model (60). As proof of concept, a recent study has shown that these siRNA tagged to magnetic nanoparticles also allow for tracking the islet cells after transplantation non-invasively by magnetic resonance imaging (MRI) (60, 61). The study demonstrated that the probe could accumulate in quantities sufficient for silencing the pro-apoptotic target genes and resulted in durable glycemic control in animals transplanted even with a marginal number of labeled islets compared to controls. Further, the nanoparticles were found to act as innovative and non-toxic reporters for imaging so islets labeled with siRNA conjugated to magnetic nanoparticles (MN-siCas-3) can be monitored *in vivo* after transplant using MRI (61). We have recently studied effects of these siRNA conjugated nanoparticles in an allogeneic baboon intra-portal islet transplantation model. We demonstrated promising protective effect on pre- labeled islets post transplantation in the preclinical large animal model (62). The safety profile to the donor with the I-K strategy is of course paramount, and minimizing the risk of inducing diabetes in a healthy patient must always be taken into account. By combining islet protocols with this promising strategy, not only may the amount of donor pancreas needed be reduced, but it may also prove to be a real time and non-invasive way of evaluating the islet mass *in vivo*.

### Xenogeneic Islet-Kidney Transplantation

Xenotransplant which is the transplant of organs between disparate species could be a potential solution to the obstacles preventing the I-K model from clinical trials. Use of a xenogeneic (e.g., pig) donor for the composite I-K transplant would allow for preparation of composite I-K’s in living donors, as well as eliminating the risk of not obtaining the sufficient islet volume required to correct diabetes in recipients without inducing diabetes in the donors. Xenogeneic tissue grafts of course would provide a near-limitless supply of tissues and organs for this purpose.

Swine would make an ideal candidate for donor tissues to be used in human xenotransplantation (63, 64). It would of course have been unreasonable to attempt xenogeneic I-K transplantation until the success of xenogeneic renal transplantation reached a stage where survivals would justify extending the procedure to a composite organ in preclinical pig-to-non-human primate models. Recently, however, investigators both in our center (65) and elsewhere (66, 67) have reported renal xenograft survivals of well over 6 months in pig-to-nonhuman primate models. In contrast to renal xenografts maintained with chronic immunosuppression which have eventually progressed to organ loss due to chronic rejection, we have achieved longer than 6 months survival of life-supporting pig kidneys utilizing the tolerance-inducing approach of vascularized thymic grafts (65) (Yamada K. et al. manuscript in preparation) in multiple baboon recipients. These animals were pig specific unresponsive and developed new T cells from the pig thymic grafts. Unfortunately, due to the size disparity between the two species the recipients were ultimately euthanized due to the development of cortical ischemia in the grafts and compartment syndrome in the small baboons caused by the growth of the pig life-supporting kidneys. Even though humans are much larger than the small baboons used in the study, and in theory should not have the same issue, the problems caused by organ growth should be addressed in future studies. Our group has currently initiated a project that combines composite I-K with vascularized thymic grafts in a pig-to-baboon model for future clinical I-K xenotransplantation.

## Conclusion

T1DM and ESRD continue to be a major source of morbidity and mortality for millions of people worldwide. In recent years major advancements in our understanding of the disease has led to significant strategies and breakthroughs for potential treatment modalities. Islet cell transplant, while once a purely experimental hypothesis has become a viable alternative to whole pancreas transplant. One strategy that is at the forefront is the islet-kidney model, in which the islet cells are allowed to pre-vascularize in the host environment for a period of time before transplant. This has been shown to avoid the initial IBMIR, as well as provide a continued physical barrier against the innate immune response in the host. Strategies aimed at minimizing post-transplant apoptosis of the islets through incubation with anti-inflammatory nanoparticles could reduce the amount of donor pancreas needed to be harvested, which would make the procedure safer and thus more attractive for potential donors. Furthermore, studies have shown that tolerizing the host by utilizing a strategy of durable chimerism may lead to long term avoidance of rejection of the graft through T cell mediated responses, which would avoid the long term immunosuppression regimens that cause the many well-known side effects and morbidities of immunosuppressive drugs.

Limitations remain concerning this highly promising model. Foremost, the kidney capsule itself has both a limited space to accommodate a high tissue volume of islet cells, as well as providing a relatively oxygen-poor environment owing to the poor blood supply. These limitations however are overcome by the strategy outlined in the review, in that metabolic exhaustion of the islet cells is avoided due to the native functioning pancreas. Furthermore, by placing the grafts under the kidney capsule makes for subsequent harvesting of the graft relatively easy. Ongoing work with xeno studies, in which the donor pool could include other species such as the miniature swine would lead to an infinite number of donor organs to be utilized.

## Author Contributions

TP and KY: Primarily wrote the review article. CS and PW: Participated in writing the review article. KY: Corresponding author. All authors contributed to the article and approved the submitted version.

## Funding

This work was supported in part by R01DK105503, R01DK105468, U19AI131474, and P01AI045897.

## Conflict of Interest

The authors declare that the research was conducted in the absence of any commercial or financial relationships that could be construed as a potential conflict of interest.

## Abbreviations

CKD, chronic kidney disease; CyA, Cyclosporine A; DCCT, Diabetes Control and Complications Trial; ESRD, End stage renal disease; IBMIR, instant blood-mediated inflammatory reaction; IDDM, insulin-dependent diabetes mellitus; I-K, composite islet-kidney; IEQ, islet equivalent; ITC, Irradiation, T cell depletion, and cyclosporine A; MHC, major histocompatibility complex; MMF, mycophenolate mofetil; MN-siCas-3, siRNA conjugated to magnetic nanoparticles; NHP, non-human primate; PBHSCTx, peripheral blood hematopoietic stem cell transplant; siRNA, small interfering RNA; SPK, combined kidney/pancreas transplant; T1DM, Type 1 diabetes mellitus; TBI, total body irradiation;TLR4, toll like receptor 4; UKPDS, UK prospective diabetes study.
